# Duplicated Collecting System with Ectopic Vaginal Implantation

**DOI:** 10.5334/jbsr.1524

**Published:** 2018-10-03

**Authors:** Maxim Behaeghe, Patrick Seynaeve, Koenraad Verstraete

**Affiliations:** 1 Ghent university, BE; 2 AZ Groeninge Kortrijk, BE

**Keywords:** Ectopic ureter, Ectopic vaginal implantation, Duplicated collecting system, Complete duplication, Ureterocele, Incontinence, Reflux

## Abstract

We present a case of a 54-year-old woman with a left-sided complete duplication of the ureter. The upper moiety drains in the proximal third of the vagina, which results in an ureterocele and urinary incontinence. The ureteral orifice of lower moiety ureter was normal. Duplication of the ureters with distal, infra-sphincteric, vaginal implantation is an uncommon congenital anomaly and a rarely seen entity in adulthood as a cause of urinary incontinence. MR colpocystodefecography showed an ureterocele in between the bladder and the rectum. Computed-tomography showed the duplicated ureters and ectopic ureter with proximal implantation on the renal upper pole and distal implantation on the proximal third of the vagina.

## Introduction

The combination of a duplicated collecting system with ectopic implantation is a rare congenital anomaly, in contrast to a duplicated collecting system, which is more common. The duplicated renal collecting system can be partial or complete, depending on a partial or full-length separation of the ureters. Although the clinical presentation can be variable, most patients develop symptoms in childhood. We present a case of a middle-aged woman with a new-onset urinary incontinence caused by the complete duplication of a ureter with the ectopic ureter draining in the proximal third of the vagina.

## Case report

A 54-year-old woman was admitted to our hospital for stress incontinence, overactive bladder and the feeling of prolapse. There was no relevant medical history. Physical examination revealed no clinical signs of prolapse. Urologic ultrasound was performed and showed a fluid-filled tubular enlargement beneath the bladder.

**Figure 1:**
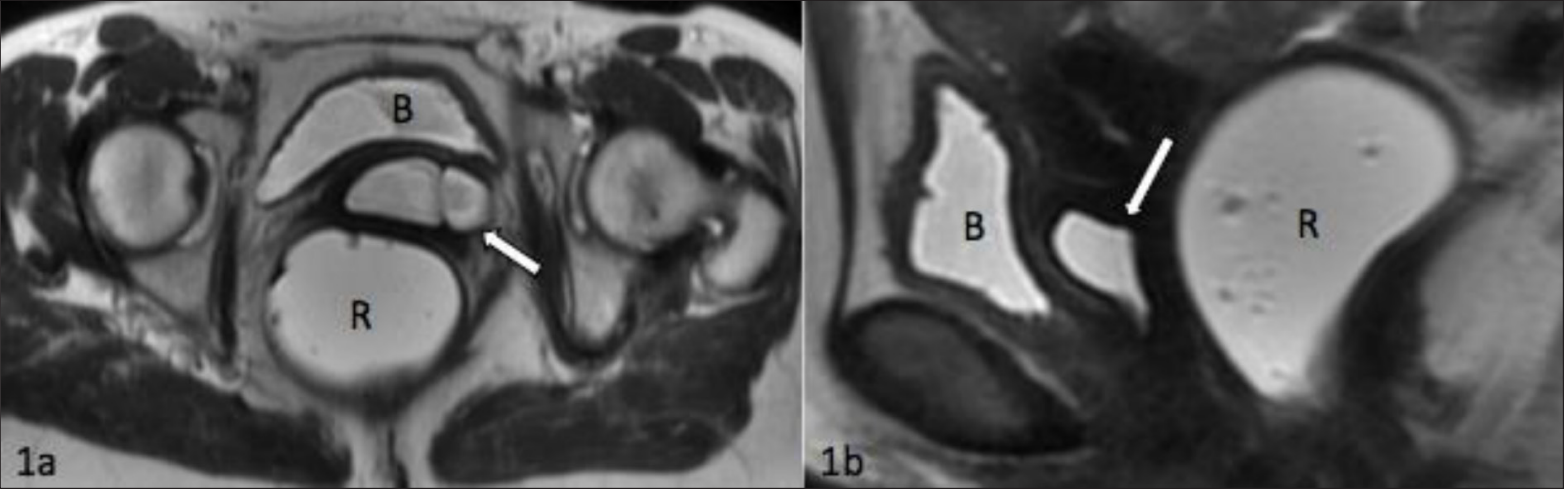
**(a, b)** Axial and sagittal view of T2 weighted MR colpocystodefecography shows a hyperintense tubular structure, ureterocele (white arrow), in the pelvic cavity in between bladder (B) and rectum (R). The rectum was filled with ultrasound gel.

**Figure 2:**
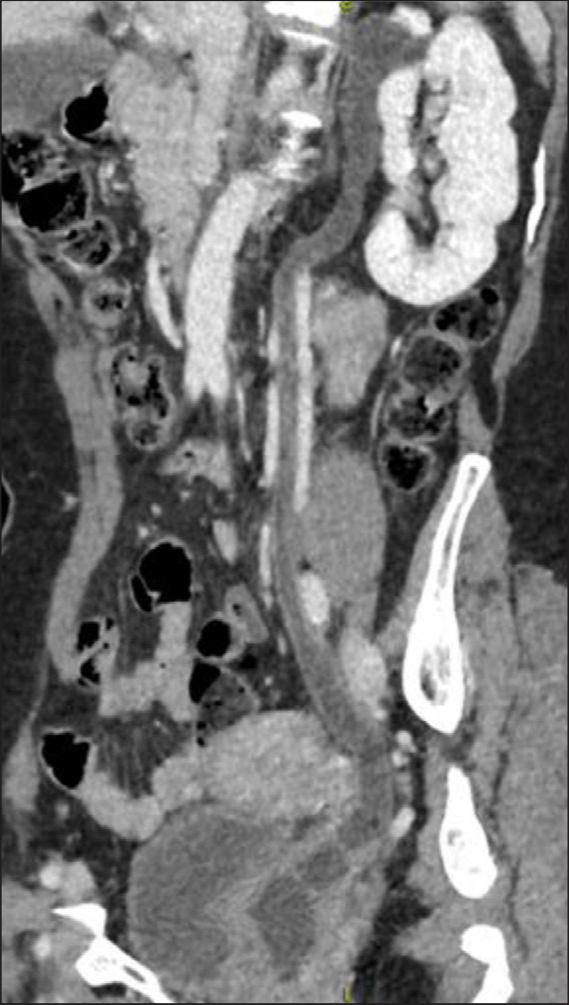
Curved reformatted image of the contrast-enhanced computed tomography shows the full-length ectopic dilated ureter and the left side with proximal implantation on the left upper renal pole and distal implantation on the proximal third of the vagina.

**Figure 3:**
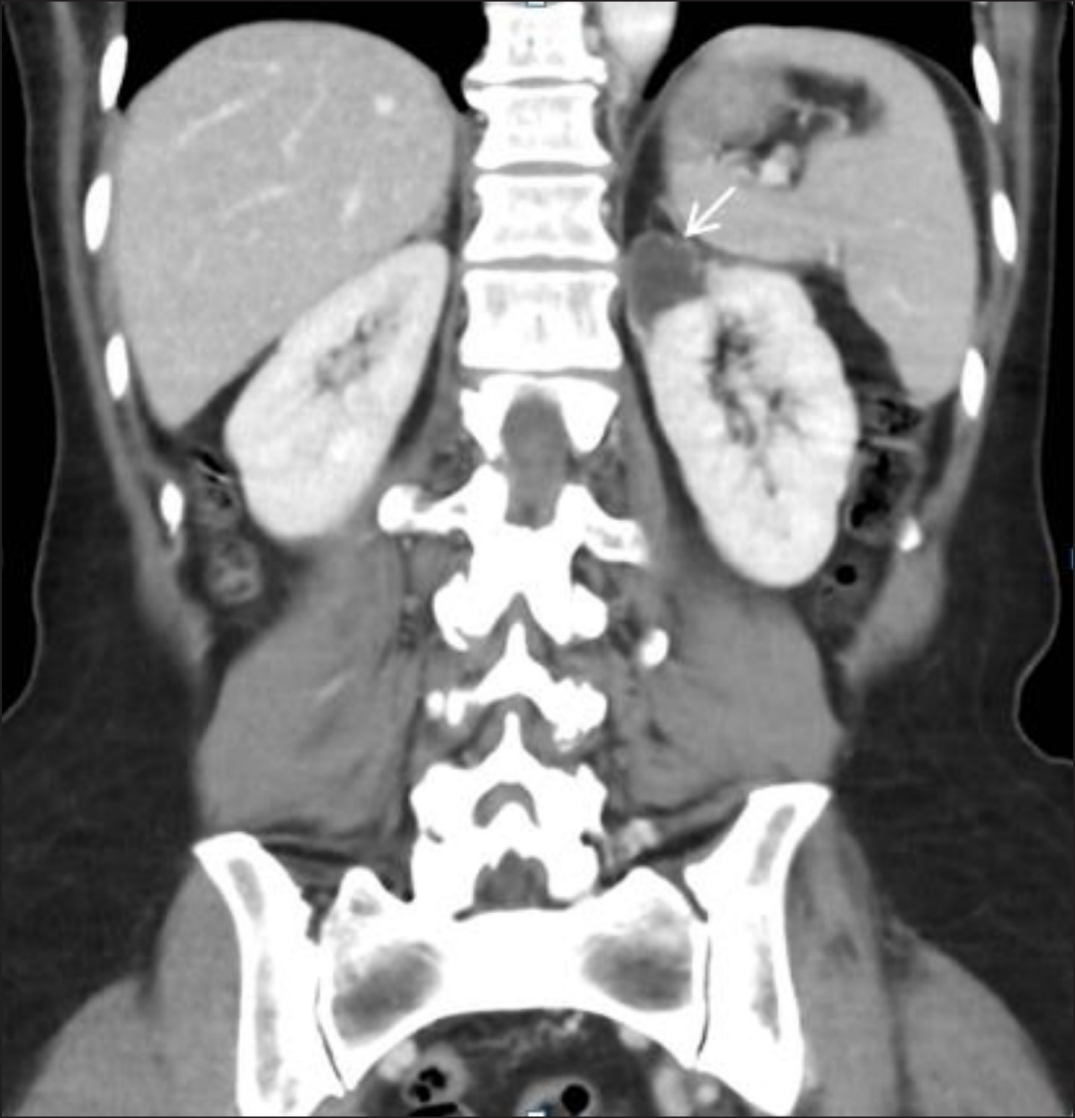
Coronal view of a contrast-enhanced computed tomography shows the upper renal moiety, which is dilated. Due to long-standing hydronephrosis in the upper pole there is a significant secondary parenchymal loss (white arrow).

MRI colpocystodefecography showed an intrapelvic fusiform enlargement (ureterocele), hyper-intense on T2-weighted MR images (Figure 1a and 1b). Computed tomography of the abdomen with intravenous contrast demonstrated a duplicated collecting system on the left side with proximal implantation on the renal upper pole, ureterocele and distal ectopic ureteral insertion on the proximal third of the vagina (Figure 2). Due to the hydronephrosis there was secondary parenchymal loss on the upper pole of the left kidney (Figure 3). The contralateral kidney and ureter were normal.

## Discussion

Duplication of the ureters is a relatively common congenital anomaly with an incidence of 0.7% in the population [1]. An ectopic ureter implantation, however, is a rare ureteral entity with an estimated incidence of 0.05–0.025% in the population [2], the majority of whom are women in up to 80% of cases [3]. During embryogenesis, the urogenital system and the reproductive system develop from the intermediate mesoderm. Duplication of the ureters occurs when two separated buds arise from a single Wolffian duct, also known as mesonephric duct, resulting in two ureters draining a single kidney. Ureteral duplication can be partial or complete, the latter being most common in 70% of the cases [4]. In this case, there was a complete ureteral duplication, which occurs when the ureteral bud arises twice (rather than splitting), resulting in one ureter ending normally into the bladder and the other being ectopic, ending in the urethra, vulval vestibule or, as in our case, the vagina [5,6]. A complete ureteral duplication is commonly associated with ectopic ureterocele, ectopic ureteral insertion and vesicoureteral reflux. The ectopic ureteral insertion at the proximal third of the vagina, distal to bladder neck and external sphincter, causes urinary incontinence and enuresis in childhood. The onset of symptoms is rarely witnessed in adulthood, which is remarkable in this case [5]. The Weigert-Meyer law can delineate the relationship of upper and lower moieties in duplicated ureters. Due to complete ureteral duplication, the upper renal moiety drains inferior to the lower ureteral moiety and ends in an ureterocele, which obstructs its own draining system and can lead to reflux in the lower ureteral moiety [7].

Diagnostic tools include ultrasound, intravenous urography, voiding cystourethrography, CT and MR imaging. Ultrasound is the imaging of choice in the initial assessment, since it is widely available, low-cost and can often detect major abnormalities, such as an ureterocele or hydronephrosis. However, small abnormalities can be missed and assessment of the complete tract or the insertion site of an ectopic ureter can be challenging. IVP and voiding urethrography are imaging techniques that can provide complimentary visualisation of the anatomical course of the ureter and its insertion site, but can be challenging to perform, especially in children. In this case, the diagnosis was made using CT scan with intravenous contrast, which showed a duplicated collecting system on the left side with proximal implantation on the renal upper pole, an ureterocele and the distal ectopic ureteral insertion on the proximal third of the vagina (Figure 2). Direct contrast injection in the dilated ureter can be indicated when intravenous computed-tomography failed to image the duplicated ureter [8]. However, the high radiation exposure is an important drawback, especially in younger patients. Unfortunately, no excretory phase was performed in our case, which could have provided valuable additional information.

In literature, MR urogram is considered to be the modality of choice, since it is able to visualise complex urogenital anomalies [9] with excellent imaging of the kidney, ureteral tract and insertion site. Furthermore, there is no risk of radiation exposure. In our case, only MR colpocystodefecography was performed, with only partial visualization of the anatomical urogenital system.

In conclusion, we presented a case of a patient with a duplicated collecting system and ectopic vaginal implantation, which is a very rare congenital anomaly. To our knowledge, this is the first case report that describes a new onset of urinary incontinence in an adult patient, caused by this urogenital abnormality. Although there are many available diagnostic tools, MRI urogram is the modality of choice, since it is able to visualize complex urogenital anomalies in detail without the risk of radiation exposure.
